# Sex Differences in Urate Handling

**DOI:** 10.3390/ijms21124269

**Published:** 2020-06-16

**Authors:** Victoria L. Halperin Kuhns, Owen M. Woodward

**Affiliations:** Department of Physiology, University of Maryland School of Medicine, Baltimore, MD 21201, USA

**Keywords:** sex differences, gout, uric acid, serum urate, ABCG2, SLC2A9, URAT1

## Abstract

Hyperuricemia, or elevated serum urate, causes urate kidney stones and gout and also increases the incidence of many other conditions including renal disease, cardiovascular disease, and metabolic syndrome. As we gain mechanistic insight into how urate contributes to human disease, a clear sex difference has emerged in the physiological regulation of urate homeostasis. This review summarizes our current understanding of urate as a disease risk factor and how being of the female sex appears protective. Further, we review the mechanisms of renal handling of urate and the significant contributions from powerful genome-wide association studies of serum urate. We also explore the role of sex in the regulation of specific renal urate transporters and the power of new animal models of hyperuricemia to inform on the role of sex and hyperuricemia in disease pathogenesis. Finally, we advocate the use of sex differences in urate handling as a potent tool in gaining a further understanding of physiological regulation of urate homeostasis and for presenting new avenues for treating the constellation of urate related pathologies.

## 1. Introduction

**Figure 1:**
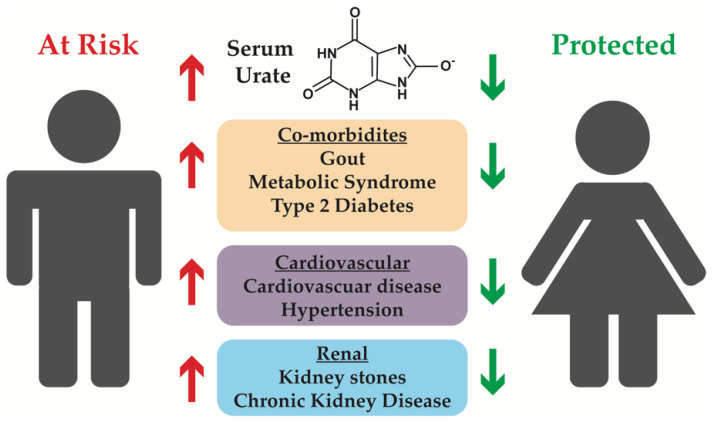
Co-morbidities associated with hyperuricemia, with an emphasis on conditions that can affect the kidneys. Males tend to have higher serum urate levels, and therefore have an increased risk of associated co-morbidities, while females have lower serum urate levels and are protected from developing associated co-morbidities.

Understanding the mechanisms of common heritable diseases has long proven to be challenging [1]. One such common human disease is gout, the most common inflammatory arthritis [2]. Gout is caused by the deposition of sodium urate (UA) crystals in the synovial fluid of joints, a process that leads to a cascade of inflammatory responses and extreme discomfort [3]. Precipitation of the weakly soluble UA occurs as the UA levels in the blood and other body fluids rise, termed hyperuricemia. Gout, or *podagra*, is one of the most well documented human diseases, recognized as early as 2460 BCE by the ancient Egyptians [4]. A staple throughout the development of the civilized world, cases were also noted by Hippocrates and Galen in ancient Greece, with a surge in cases in the 17th and 18th centuries [4] during the industrial revolution, commiserate with rising wealth across the western world. Today, the prevalence of gout is roughly 4% of the population of the United States, Europe, and Southeast Asia [5]. Interestingly, recent research has found that gout is only one of many potential diseases caused or contributed to by hyperuricemia (HUA); HUA is also an independent risk factor for additional pathologies including renal diseases, cardiovascular disease, hypertension, and metabolic syndrome [5,6,7] (Figure 1). As this list of diseases continues to grow, so does the critical need for a deeper understanding of not only how we regulate UA homeostasis, but also the causes of disruptions of these processes.

A mechanistic picture of the effectors of UA homeostasis has emerged, yet we lack an understanding of how these proteins are regulated. New tools are needed to examine this problem to provide relief for the large and growing population of hyperuricemic individuals [1]. Additionally, new work has reinforced the importance of an ancient observation [4], the male sex is a significant risk factor for hyperuricemia and gout [8,9], where men are up to four times more likely to be affected than women [5]. This observation has now been coupled to investigations of differences in UA handling in the kidney, as well as differential effects of pathogenic variants in UA transporter genes, presenting the potential to use sex differences in UA handling as a powerful comparative analysis revealing how these systems are regulated. Here, we review sex differences in UA handling to explore how the female sex may be protective against the development of UA related diseases and to illustrate the power of using sex differences as a tool.

## 2. Urate as a Risk Factor in Disease

HUA is clinically defined as elevated serum UA (SUA, >6mg/dL), which increases the risk of the precipitation of weakly soluble UA. Increased UA leads to precipitation of monosodium UA crystals, which can cause UA kidney stones and gout. The number of affected individuals continues to climb with current estimates being approximately 47.2 million HUA individuals in the United States; roughly 27.9 million individuals have severe HUA (>7mg/dL), with men being affected five times more often than women [6]. Similar results have been observed in China, with an overall HUA prevalence of 13.3%, with striking differences in prevalence by sex, with 19.4% in men and only 7.9% in women [10]. However, the risk of the development of HUA increases roughly four-fold for women after menopause, and postmenopausal hormone replacement therapy reduces this risk [11,12,13], providing evidence that female hormones may contribute to the protection from HUA. 

HUA is just one of many conditions that have observable sex differences, as differing pathologies based on sex have been observed in a variety of fields [14,15,16,17] including cardiovascular [18,19,20,21], neurological [22,23,24,25], immunological [26,27,28], and renal diseases [29,30,31,32,33]. The architecture of the female kidney is likely distinct from that of the male kidney [34], given women have a lower blood pressure than men [35], women are less likely to develop acute kidney injury than men [36,37], women demonstrate improved tolerance to renal ischemia [38,39], and women are protected from renal and cardiovascular disease before menopause as compared to men [40,41]. A recent study determined that females with chronic kidney disease (CKD) had a slower decline in glomerular filtration rate (GFR), lower risk of progression to end-stage renal disease (ERSD), and a lower risk of death compared to age-matched men with similar mild-to-moderate CKD [42]. In diabetic kidney disease, men tended to demonstrate renal complications roughly 10 years earlier than women [40], while sex differences in obesity-related kidney disease [43,44,45] demonstrated that female sex hormones may safeguard against worsening pathology [45]. Thus, being female may have a protective effect on the kidney, preventing women from developing the more severe phenotypes observed in men. All these studies illustrate the need for greater emphasis on the idea of sex as a biological variable [46,47,48], going beyond merely looking at sex differences, and instead examining the influence of sex on various physiological and pathophysiological pathways [49] to help elucidate underlying mechanisms. 

Although the overall kidney function seems to be improved in females when compared to males, once female kidney function does begin to decline, the resulting pathologies can be more extreme than in males, in part due to the fact that this decline is likely to occur later in life in women. As kidney function declines, SUA levels tend to increase, and increases have been reported to be associated with all-cause and cardiovascular mortality in a dose-dependent manner. Thus, as UA levels increase, so does the risk of developing other conditions, including CKD, hypertension, and diabetes mellitus [50,51,52,53,54,55], especially in women [12,56]. Table 1 summarizes data that compares the comorbidities of individuals who have low SUA with individuals who have high SUA, stratified by sex. HUA increases the risk of developing CKD for both men and women and increases the likelihood of progression to ESRD in both sexes, yet this risk is much higher in HUA females than HUA males [50]. Similarly, the incidence of CKD increases two-fold to six-fold higher in HUA females than HUA males [51,52], demonstrating that once females have lost the benefit of low SUA, renal health is more likely to decline. Males and females with both HUA and CKD had a higher incidence of left ventricular hypertrophy and hypertension, however, this association was only significant in females [53]. HUA also increases the overall risk for hypertension for both sexes, but once again females with HUA are more likely to develop hypertension than HUA males compared to non-HUA controls [51,54,57]. Finally, females have a higher incidence of type 2 diabetes mellitus than males [52], yet HUA females are even more likely to develop diabetes than HUA males [54], further emphasizing that an increase in SUA can be more detrimental to females than males. However, since HUA is only one of several pathologies to affect people later in life, the role of UA in the biogenesis of the underlying pathology is difficult to tease out, demonstrating the critical need for additional studies.

Taken together, this evidence implies that both biological sex and SUA level affect the kidney, in that the interaction of these two variables may influence renal function. The female sex must somehow affect the balance of UA levels, leading to a decrease in SUA when compared to males. Unraveling the mechanism behind this effect could lead to a better understanding of the underlying physiology and improved treatment for HUA. However, UA handling in the kidney is an incredibly complicated process, the details of which have yet to be fully elucidated.

## 3. Urate Homeostasis

Renal UA handling involves multiple transporter proteins. UA is the protonated form of uric acid and enters the circulation as the terminal metabolite of purine metabolism in humans and the other great apes. It is produced from the degradation of purine nucleotides and amino acids, mediated by xanthine oxidase. Higher-order primates have lost the activity of the enzyme uricase, which further metabolizes UA to the much more soluble allantoin [58]. Loss of uricase gene function in the ape lineage supports the idea of a possible selective advantage of increased SUA [59,60]. Contributing to this complicated process is the fact that the physiological concentrations of UA occupy a wide range, which is higher in men (3.5 to 7.2 mg/dL) than in pre-menopausal women (2.6 to 6.0 mg/dL) [61]. Interestingly, concentrations in post-menopausal women increase to levels observed in men [62]. Since UA is weakly soluble, high concentrations (>6 mg/dL) can lead to precipitation and crystal formation resulting in UA kidney stones. These crystals can also precipitate in joints and synovial fluid causing gout [6].

Of the UA excretion, 70% is mediated by the kidney and 30% by extrarenal pathways including the gut and the liver [63,64,65]. A delicate balance between secretion and reabsorption exists in the kidney to maintain UA homeostasis. UA is freely filtered at the renal glomerulus, then reabsorbed, actively secreted, and reabsorbed again in the proximal tubule [66,67,68,69]. Several proteins have been identified as UA transporters based primarily on in vitro studies demonstrating UA affinity. Based on in vivo renal tubule expression, the initial reabsorption of 95% of the initial filtered UA load may occur via organic anion transporter (OAT) family proteins, including OAT1 (encoded by *SLC22A6*), OAT3 (encoded by *SLC22A8*), OAT4 (encoded by *SLC22A11*), and OAT10 (encoded by *SLC22A13*), as well as SLC2A9 (also called GLUT9) [70,71,72,73,74]. Approximately 50% of the initial filtered load is then actively secreted back into the tubular lumen, primarily where ABCG2, NPT1 (encoded by *SLC17A1*), and NPT4 (encoded by *SLC17A3*) are expressed [75,76,77,78,79,80]. Reabsorption of another 40% of the filtered UA occurs downstream of the secretion in the S3 segment of the proximal tubule, where expression of URAT1 (encoded by *SLC22A12*) and SLC2A9 have been reported [71,81], resulting in a fractional excretion (FEUA) of 4%–8% of the initial filtered load. This somewhat controversial [69] and complicated mechanism of filtration, reabsorption, secretion, and a second round of reabsorption demonstrates that the kidney spends an exorbitant amount of energy fueling these transporters in an effort to carefully regulate UA excretion from the kidney for reasons yet to be fully understood.

Functional data of these and other [82,83,84] transporters demonstrate that these proteins can transport UA. However, whether or not transport of UA occurs in the renal tubules by these particular transporters remains unclear. The next logical step would be to explore genetic perturbations in these transporters to determine if those alterations affect SUA levels. Thus, exploring human genetics and genetic variations in the form of genome-wide association studies (GWAS) can be a powerful tool for understanding which genes are most important to UA handling. GWAS use common single nucleotide polymorphisms (SNPs) that exist in a given population as signposts for genomic space that correlate with a given condition [1], including SUA levels. The larger the study population, the higher the genomic resolution to identify regions of interest that associate with altered SUA. Once these genomic regions have been identified, additional analyses can then be performed to identify those genes most likely to underlie the associated SNP, in some cases identifying novel causal variants that contribute to the development of HUA. 

## 4. Genetics of Hyperuricemia

SUA levels display a strong heritable component, estimated between 40%–70% [85,86,87]. The first gene associated with UA transport in humans was *SLC22A12* [81]. A labor-intensive comparative and candidate based cloning approach identified the locus of *SLC22A12* as potentially harboring the key gene. The resulting protein product, URAT1, proved to be a kidney-specific UA transporter, and variants were found in a Japanese family with significant hypouricemia [81]. *SLC22A12* variants causing hypouricemia have been reported at high frequencies in Japanese [88], Korean [89], and European Roma [90] populations, reinforcing the importance of URAT1 function on SUA levels. Sex differences, however, have not been reported in the context of hypouricemia. Subsequent work proved that other members of the SLC22A gene family (the OATs) proved to have UA affinity [70,91], but their physiological relevance proved difficult to substantiate [69]. Progress was only made in identifying other key UA related genes with the advent of GWAS, allowing researchers to use an unbiased approach to discover genes and gene products that are involved in specific phenotypes like SUA levels or gout risk [85,86,87,92]. Because of the ease of measurement and the availability of serum samples from large cohorts of individuals, GWAS for SUA have played a driving role in demonstrating the incredible power of the tool to find gene networks or even specific transporter genes playing a significant role in UA homeostasis in humans [92,93,94]. The GWAS published on UA over the past decade [94,95,96,97,98,99,100,101,102] have been progressively increasing genomic resolution by increasing the number of individuals used, thus genes with smaller and smaller contributions to the SUA phenotype were able to be resolved. A recent effort, the largest yet, performed by Tin et al. [101], explored variants in 457,690 individuals of various ethnic backgrounds and found 147 new SNPs and replicated 36 previously reported SNPs that associate with SUA, all of which were further replicated in an independent cohort of 334,880 individuals. As part of their analysis, Tin et al. also attempted to identify common variants causal for alterations in UA levels. Emphasis was placed on missense variants with the most significant combined annotation dependent depletion Phred score, revealing six potential causal variants. These potential causal variants included SNPs in the well-established UA transporter gene *ABCG2* [76], as well as in the transcription factor genes *HNF1A* and *HNF4A* [101]. Investigators demonstrated the causative nature of the *HNF4A* variant by showing that it altered the ability of its protein product HNF4α to directly regulate trans-activation of *ABCG2* [101]. 

Only 6 of the 183 loci in that same study demonstrated sex-specific effect differences, including *SLC2A9*, *ABCG2*, *CAPN1*, *GCKR*, *IDH2*, and *SLC22A12* [101]. However, of these six, only *SLC2A9* and *ABCG2* were found to have significant associations in the more stringent genome-wide test for SNP effects on UA levels based on sex, confirming previously established findings [92,93,94,103,104]. Table 2 displays a current list of SNPs that have reported sex differences in genes that associate with SUA levels confirmed by multiple GWAS. Initial reports demonstrate that *SLC2A9* had a stronger association with lower SUA levels in females, whereas *ABCG2* had a stronger association with higher SUA levels in males, demonstrating that regulation and/or activity of these transporters may be influenced by factors that contribute to biological sex [92]. Significant association with HUA associated gout risk has been reported in males, but not females, for several other transporter genes (*SLC16A9*, *SLC17A1*, *SLC22A11*, and *SLC22A12*), as well as UA transporter “keystone” scaffold protein encoded by *PDZK1* [100]. Additional sex differences in these genes are detailed in Table 2, as well as some of the UA associated transcription factors *HNF4A* and *HNF4G* [105,106,107]. Understanding the interactions between these genes will lead to a more accurate model of renal UA handling, revealing those genes with the most significant contributions to UA levels. 

The picture of the genetic architecture emerging is one of two tiers, first a base composed of a large number of genes that provide a significant but small contribution to the variance in SUA, and then a top tier with the three critical UA transporter genes: *ABCG2*, *SLC2A9*, and *SLC22A12*. As mentioned above, variants in *ABCG2* and *SLC2A9* have been demonstrated to exert sex differences in effect size, but not *SLC22A12*. This may speak to one of the limitations of GWAS, such that these studies can only use common variants present in high enough frequencies to be detected, leaving rare but potential associated and causal variants to remain undetected in these studies. To address this, recent work that used whole-exome sequencing (WES) to probe for rare variants that contribute to SUA variance found numerous rare variants including two in the *SLC22A12* gene (rs150255373 encoding R325W and rs147647315 encoding R434H) that had large effect sizes [108]. The lack of common variants in *SLC22A12* may have contributed to its under-reporting of sex effect. Additionally, *SLC22A12* expression was found to be higher in males over the age of 50 compared to females of a similar age, yet *SLC22A12* expression was the highest in females <50 as reported in a smaller transporter targeted study [109]. Increased *SLC22A12* expression could indicate an increase in UA reabsorption, yet females under the age of 50 have some of the lowest levels of SUA of all populations sampled, implying that the reabsorption of UA by URAT1 may not be the primary contributor to SUA in females. This primary contribution is much more likely due to commiserate changes in other UA transporters. 

### 4.1. SLC2A9

Not coincidentally, the two genes variants with the largest sex-specific effect differences are also the two with the common variants contributing the most to SUA variability (*SLC2A9*) and gout risk (*ABCG2*) for both adult [125] and pediatric-onset [126]. SLC2A9 is a high affinity UA transporter and one of two transporters that are primarily responsible for the reabsorption of UA [71,104]. A recent WES association study revealed dozens of variants in *SLC2A9*, including 90 rare variants that associated with SUA, and an additional number of common variants that map onto important functional regions of the protein [108]. Interestingly, SNPs of *SLC2A9* are associated with both decreased SUA [92,94,127,128] and increased SUA [97], dependent upon the SNP. Of identified *SLC2A9* SNPs associated with a decrease in SUA, this decrease was much greater in women than in men (rs7442295 yielded −0.503 mg/dL in women, while only −0.202 mg/dL in men), with a higher heritability component in females than in males in European populations [104]. These results were further confirmed in a case-control study, where the partial loss of function variant of *SLC2A9* contributed to a lowering of UA levels between 0.30–0.35 mg/dL or 5%–6% of all populations sampled, with a much stronger association in women [129]. These results support the idea that SLC2A9 mediated reabsorption is an important mechanism of SUA regulation in females. 

### 4.2. ABCG2

ABCG2, the other major transporter, transports UA out of the cell [76], and is expressed in the kidney, liver, and small intestine, as well as the blood-brain and blood-placental barriers, and in mammary epithelial cells [125]. It is expressed in the renal proximal tubule in both humans and mice [130] where it plays an important role in UA secretion. Several variants in *ABCG2* have been associated with SUA levels, the most well-characterized of which is rs2231142, which encodes the missense mutation Q141K. This risk allele is common among several populations ranging from 3% in populations of African ancestry, 11% in those of European ancestry, and up to 31% in East Asian populations [131]. The Q141K variant confers a partial loss of function, characterized by a loss of transporter activity and reduced stability of the N-terminal nucleotide-binding domain leading to decreased protein abundance at the cell surface via increased endoplasmic-reticulum-associated-degradation [94]. However, in humans, possessing the Q141K variant did not translate to the predicted significant changes in FEUA [94,132,133,134].

**Figure 2:**
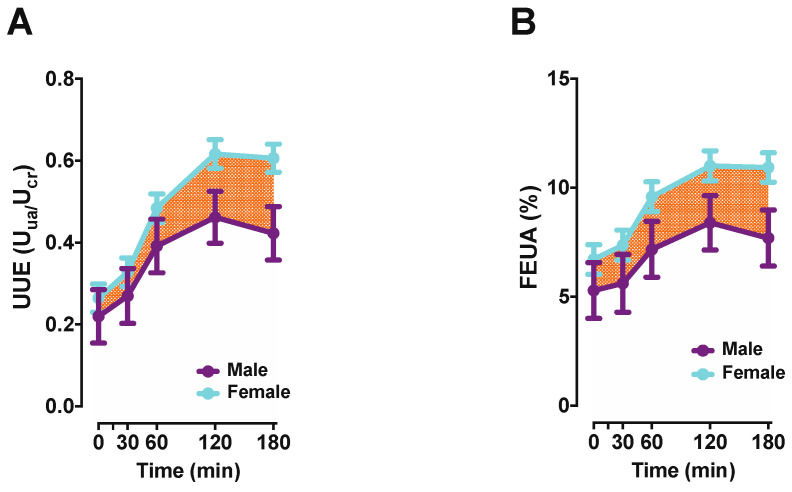
Effect of sex on urinary urate excretion (UUE) (**A**), and fractional excretion of urate (FEUA) (**B**) in participants following inosine load (79 participants; ± SD of mean). Statistical significance evaluated by a two-tailed ANCOVA, adjusted for age, ancestry, and BMI. ACOVA *P*_(time)_ < 0.0001, *P*_(sex)_ < 0.0001 for Panels (**A**,**B**), ACOVA *P*_(sex*time)_ = 0.038 (**A**) and *P*_(sex*time)_ = 0.45 (**B**). Adapted from Hoque et al., Nature Communications [130] as allowed under the Creative Commons Attribution 4.0 International License (http://creativecommons.org/licenses/by/4.0/).

A recent study attempted to better understand the role of ABCG2 in the kidney and the impact of the Q141K variant on kidney function in a relatively normal young population [130]. The participants were challenged with oral inosine to acutely increase SUA levels. Participants with the Q141K variant had significantly higher SUA levels at baseline and throughout, yet a comparison of the FEUA between genotypes revealed no differences. These findings suggest that changes in FEUA did not contribute significantly to increases in SUA [130] in the Q141K individuals. Instead, the Q141K individuals lost a significant portion of the extra-renal contribution to SUA variance, implicating a strong role for ABCG2 in intestinal UA secretion, but unfortunately not clarifying the role, if any, of ABCG2 in the kidney. Interestingly, that same study examined non-risk allele carriers stratified by sex and found females to have a lower SUA in response to inosine, with higher FEUA and higher urinary UA excretion (Figure 2). These data implied that the female kidney responded differently to the higher UA load, but also better compensated for this higher UA load by increasing excretion.

## 5. Regulation of UA Transporters

### 5.1. Transcription Factors

The effectors of UA homeostasis, the transporters, are an obvious mechanism for explaining sex differences but they exist downstream of other differentially regulated pathways, such as transcription factors. While several transcription factors have been implicated in the regulation of UA transporters, for example, HIF-1α and Nrf2 can regulate *ABCG2* expression [135,136,137], these transcription factors do not associate with changes in SUA levels in reported GWAS. A more appealing and probable target would be a transcription factor that does associate with SUA levels in humans. Such a target was recently revealed, where transcription factor genes *HNF1A* (encoding HNF1α) and *HNF4A* (encoding HNF4α) had significant associations with SUA [101]. Targeted studies have shown that the transcription of UA transporters can be directly regulated by these HNF family transcription factors [138]. Specifically, HNF1α can regulate expression of *SLC22A12* [139] and important scaffold gene *PDZK1* [140], while HNF4α has been reported to trans-activate *SLC2A9* [122] and *ABCG2* [101], and also facilitates the proper expression of *PDZK1* [141,142]. Index SNPs in *HNF1A* demonstrated a borderline significant increase in SUA and a decrease in FEUA in women, but not men [94]. Both *HNF1A* and *HNF4A* may be influenced by sex hormones [105]. The *HNF4A* gene has been reported to have estrogen receptor (ER) enhancer elements [143], and the HNF4α protein may interact with ER-α to affect the transcription of target genes [144]. In humans, pancreatic islets demonstrate differential methylation patters of the *HNF4A* locus between males and females [106], supporting evidence that HNF4α may be associated with islet function [123]. Relatedly, cultured primary hepatocytes isolated from females have reported roughly two-fold higher non-sex hormone-stimulated activation of HNF4α than those isolated from males [107], demonstrating that HNF4α activity may be increased in females when compared to males. *HNF4A* has been further characterized in mice, in which male HNF4α-null mice demonstrated 1000 more affected genes than females [145]. Thus, since HNF1α and HNF4α affect UA handling machinery and may be affected by sex hormones, these transcription factors are promising candidates for further study.

### 5.2. Estrogen

To explore the possible role of sex hormones in regulating UA transporters and more generally UA levels, an early study examined the effects of oral administration of synthetic sex hormones on both women and men [146]. The study demonstrated an inverse relationship between SUA and administered exogenous estrogen, as well as an increase in urinary UA. Similar effects were observed when post-menopausal women received progesterone. Additional studies demonstrated changes in SUA during ovulation in pre-menopausal women [147], with peak SUA levels occurring during the follicular phase [148] when estrogen levels are lowest. Furthermore, SUA was positively correlated with follicle stimulating hormone and inversely associated with estradiol and progesterone [148]. Another study examined the effects of hormone therapy on transsexual participants, demonstrating a decrease in SUA in male-to-female (MTF) participants, and an increase in SUA in female-to-male (FTM) participants after one year of cross-sex hormone administration. Furthermore, baseline FEUA was higher in FTM subjects and significantly decreased after the loss of estrogen, while MTF subjects demonstrated an increase in FEUA with the addition of estrogen [149]. These studies provide strong evidence for the role of female sex hormones in regulating FEUA, which can strongly contribute to SUA. Finally, as female sex hormone levels decrease after menopause, SUA increases, but this increase in SUA can be mitigated by hormone replacement therapy [12]. This evidence further reinforces that the female sex hormones may be regulating UA handling machinery, at either the transcriptional or post-transcriptional levels. Estrogen could explain the observed sex differences in UA associated UA transporters. Therefore, understanding the role of estrogen in regulating UA transporters is critical in unraveling the complex mechanisms of UA handling.

Estrogen has been reported to have direct effects on sex-associated UA transporters. For example, ER binding sites have been identified in the promoter region of *ABCG2* [150], implying *ABCG2* can be transcriptionally regulated by estrogen. Additional reports have demonstrated that ABCG2 protein expression is down-regulated by estrogen [151], possibly through proteasome activation via ER-β induced PTEN/PI3K/AKT signaling [152]. Based on these studies, increased estrogen could decrease ABCG2 expression, which would decrease secretion, leading to an increase in SUA. This seems to be in conflict with the fact that females have lower SUA levels, demonstrating that in vitro models may have limited utility regarding ABCG2 regulation. Combinatorial signaling through ER-α and ER-β may also induce internalization of ABCG2 protein at the plasma membrane, decreasing ABCG2 activity, as observed in the blood-brain-barrier of mice [152]. This study provides some evidence of estrogen-mediated post-transcriptional regulation of ABCG2, yet further studies are required to elucidate the role of estrogen regulation of ABCG2 in the kidney. Similarly, estrogen has also been shown to downregulate SLC2A9 at the post-transcriptional level through ER-β induced proteasomal activation [153]. Decreased SUA levels in females could be explained as ER-β signaling causing a greater decrease in SLC2A9 than ABCG2, resulting in a greater decrease in the reabsorption of UA, leading to a higher FEUA, as seen in females, however, this requires additional study.

## 6. Modeling HUA Associated Sex Differences

### 6.1. Established Mouse Models

The complexity of human physiology and the heterogeneous nature of human populations make mechanistic studies difficult. Thus, effective model systems are needed to further progress our understanding of UA homeostasis and the role of sex in its regulation. Animal models are widely used to provide insights into human disease, but the specific differences in human and rodent UA physiology present additional hurdles [154,155]. One hurdle that needs to be potentially overcome when using mice to model HUA is that mice have a functional *Uox* gene, resulting in the production of the uricase enzyme, which then breaks down UA into allantoin. To remove this hurdle, a mouse with genetic ablation of the *Uox* gene was first described in 1994 [156], resulting in increased SUA levels in both males and females. Unfortunately, as much as 40% of the animals died within the first five weeks of life, with even the survivors displaying significantly increased incidence of UA crystal deposition in the kidney and other tissues, limiting the utility as a model of hyperuricemia. Therefore, additional mouse models that develop HUA by slightly different mechanisms are required for further study.

Other key UA transporter genes have been targeted in attempts to raise the SUA levels in mice, including URAT1, SLC2A9, and ABCG2. *SLC22A12* has been knocked-out on both a mixed [157] and a pure C57Bl/6J background [70]. Both *SLC22A12* knock-out models are grossly physiologically normal, with either an increased urinary UA to creatinine ration along with higher FEUA [158] or increased urinary UA excretion with no apparent differences in SUA [70]. Whole-body knock-outs of SLC2A9 [159] demonstrated HUA, as well as nephropathy with UA crystals, hyperuricosuria, polyuria, and increased water intake. Interestingly, sex differences in FEUA were observed in these animals, corroborating the predicted sex differences from the genetic studies in humans. Sex differences in FEUA were lost in a liver-specific *SLC2A9* knock-out and there were no sex differences reported in the intestinal enterocyte-specific knock-out of *SLC2A9* [160]. Therefore, like the *SLC22A12* knock-out mice, *SLC2A9* knock-out mice have limited use when exploring sex differences in HUA.

### 6.2. ABCG2-Q140K Mouse Model

In contrast, a new HUA mouse model produced by inserting a human ABCG2 variant (Q140K; orthologous to the human Q141K), not only accurately recapitulates renal and gut UA transport defects observed in humans possessing the Q141K variant, but also results in profound sex differences in how the deleterious ABCG2 variant effects function. The mouse model was created in an effort to better understand if ABCG2 played a significant role in kidney UA excretion and the deleterious effects of the Q140K variant. The model demonstrated that ABCG2 is a key secretory transporter in the kidney and small intestines, however, while the Q140K variant was extremely deleterious in the intestines, it surprisingly had only a small effect on ABCG2 abundance and function in the kidney. This discrepancy in phenotypes appeared to explain why humans with the Q141K variant are at high risk for HUA: the mutant protein has little effect on transporter function in the kidney, thus differences in observed FEUA are small or are absent, yet the Q141K variant produces a profound loss of intestinal secretion of UA, driving increases in SUA [130].

**Figure 3:**
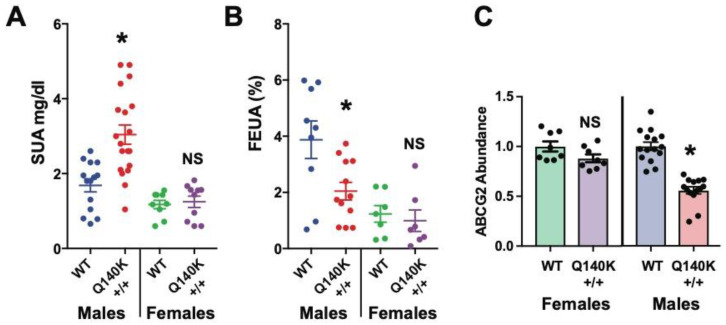
Female metabolic comparisons and ABCG2 abundance in ABCG2-Q140K mice. (**A**) Serum urate levels for male and female mice: WT and Q140K+/+ animals (WT females *n* = 9; Q140K+/+ *n* = 10; *p* = 0.7113, two-tailed Student’s t-test; males, *n* = 14 WT, and *n* = 19 Q140K+/+; * *p* < 0.0001, ± SEM). (**B**) Fractional excretion of urate (FEUA) measurements from female WT (*n* = 7) and female Q140K+/+ (*n* = 7, *p* = 0.6263, two-tailed Student’s t-test; males, *n* = 10 WT, and *n* = 12 Q140K+/+; * *p* = 0.0196, ± SEM). (**C**) Summary data from Western blots of total kidney homogenate from female WT (*n* = 8) and female Q140K+/+ (*n* = 8) mice showed no change in abundance of the Q140K+/+ protein (*p* = 0.0835, two-tailed Student’s t-test. (Males, *n* = 15 WT, and *n* = 14 Q140K+/+; * *p* < 0.0001, ± SEM). NS = non-significant. Adapted from Hoque et al., Nature Communications [130] as allowed under the Creative Commons Attribution 4.0 International License (http://creativecommons.org/licenses/by/4.0/).

A second startling finding from the ABCG2-Q140K mouse model was that the female Q140K mice did not show any phenotypes (Figure 3). The female Q140K mice had unaltered SUA, FEUA, and even unaltered renal ABCG2 protein abundance [130], suggesting these mice were representative of the sex differences observed in humans associated with HUA, and therefore could prove to be the most useful model to date. These sex differences may be due, at least in part, to the fact that ABCG2 can be regulated by estrogen [150,151,152], as hypothesized in humans. Comparing transcriptional profiles and post-translational modifications in the HUA male Q140K mice against the non-HUA Q140K female mice may reveal genes or signaling cascades critical for the regulation of UA handling in the kidney. Luckily, these further studies are now possible.

With the right in vivo models, a number of important questions are possible to address. Since increases in SUA are associated with a myriad of other pathologies, including CKD, hypertension, and diabetes, it is difficult to determine which is causal and which is the co-morbidity. In the case of CKD, can HUA promote CKD, and will amelioration of SUA levels improve the prognosis for CKD? How does biological sex potentially contribute to this relationship? There is emerging evidence that treatment of SUA levels in patients with CKD can in fact improve CKD progression in patients receiving UA lowering therapy [161,162]. In order to better answer these questions, improved models are required. An ideal model would most closely mimic the human condition, with a slower onset of increased SUA as the animals age, and distinct differences in phenotypes between male and female animals, in which females demonstrate milder symptoms. The humanized ABCG2-Q140K mouse model [130] serves as an excellent jumping-off point to begin comparisons, as these mice recapitulate the human pathophysiology and the sex differences phenotypes.

## 7. Conclusions

Female sex hormones, specifically estrogen, may play a role in the regulation of expression or activity of UA transporters, specifically ABCG2 and SLC2A9. This is emphasized by the fact that females may have differences in renal UA transporter expression, localization, or activity. Estrogen could mediate either direct transcriptional regulation of the transporter genes, or activate transporter specific transcription factors, including HNF4α. Observable differences in how transporter transcription and post-translational modification are regulated between the sexes may reveal significant information about these regulatory pathways and provide targets for therapy. Thus, using sex as a biological variable may provide key insights into understanding UA handling, elucidating underlying physiological regulatory mechanisms, and underscoring a critical need for future studies.

## Figures and Tables

**Table 1:** Sex differences in HUA associated phenotypes.

Study [Ref]	Non-HUA: *N*	SUA (mg/dL)	Non-HUA: Pathology	HUA: *N*	SUA (mg/dL)	HUA: Pathology	Increase with HUA
**Kidney Disease**							
ESRD: Chang HY, et al. 2010 [50]	**Men**: 2320	<5	10%	1825	≥7	34%	3.4-fold
	**Women**: 4248	<4	9%	1879	≥6	47%	5.2-fold
CKD: Kuwabara M, et al. 2017 [51]	**Men**: 1582	≤7.0	12.4%	282	>7.0	17.4%	1.4-fold
	**Women**: 2902	<6.0	4.8%	133	≥6.0	16.5%	3.4-fold
CKD: Redon P, et al. 2019 [52]	**Men**: 1238	<6	2%	381	>7	8%	4-fold
	**Women**: 1142	<5	1%	445	>6	6%	6-fold
**Hypertension ^1^**							
Yoshitomi R, et al. 2013 [53] (CKD patients)	**Men**: 29	<6	66%	29	>8	100%	1.5-fold
	**Women**: 23	<5	30%	27	>7	93%	3.1-fold
Kuwabara M, et al. 2017 [51]	**Men**: 1582	≤7.0	9.2%	282	>7.0	14.2%	1.5-fold
	**Women**: 2902	<6.0	10.0%	133	≥6.0	22.6%	2.3-fold
Lin, et al. 2020 [57] (BP > 140/90)	**Men**: 4150	<7	8.3%	2588	>7	12.3%	1.5-fold
	**Women**: 676	<5.7	1.3%	90	>5.7	5.6%	4.3-fold
**Diabetes Mellitus (Type 2)**							
Kuwabara M, et al. 2017 [51]	**Men**: 1582	≤7.0	1.6%	282	>7.0	1.4%	No change
	**Women**: 2902	<6.0	0.5%	133	≥6.0	2.3%	4.6-fold
Yamada T, et al. 2011 [54]	**Men**: 1561	<5.2	7.2%	1536	≥6.7	10.5%	1.5-fold
	**Women**: 1543	<3.7	2.2%	1238	≥4.8	7.1%	3.2-fold
Redon P, et al. 2019 [52]	**Men**: 1238	<6	25%	381	>7	34%	1.4-fold
	**Women**: 1142	<5	31%	445	>6	39%	1.3-fold

^1^ Hypertension defined as >130/80 mmHg unless otherwise specified; ERSD: end stage renal disease; CKD: chronic kidney disease; SBP: systolic blood pressure; DBP: diastolic blood pressure; BP: blood pressure.

**Table 2:** Reported sex differences in UA associated transporters and regulators involved in UA handling.

Gene	Protein	Description/Role	SNP Associated Sex Differences	Ref ^1^
*ABCG2*	ABCG2	UA secretion in the kidney, major UA transporter in the intestine and liver [76,110,111]	• SNP rs2231142 is reported to have gene–sex interactions in European adults, demonstrating a stronger interaction with a greater effect in men than in women [93,100], increasing SUA 0.270 mg/dL in men and only 0.181 mg/dL in women [94] • SNP rs2199936 similarly demonstrates a greater effect size in European men than in women [93]	[82,92,93,94,95,96,97,98,99,100,101,108,112]
*SLC2A9*	GLUT9	Glucose, fructose transporter, also involved in UA reabsorption [104,113]	• SNP rs734553 is most significantly associated with SUA in European men, while intron SNP rs12498742 is most significantly associated with SUA in European women [93] • SNP rs7442295 yielded a decrease in SUA of –0.503 mg/dL in women, while only –0.202 mg/dL in men, with a higher heritability component seen in European women [104] • SNP rs11722228 associates with UA levels significantly in Chinese men but not women [114]	[82,92,93,94,95,96,98,99,100,101,102,108,112]
*SLC16A9*	MCT9	pH-dependent, sodium-sensitive monocarboxylate transporter, with a reported affinity for creatine [115]. No evidence for direct UA transport.	• Missense variant rs2242206 (K258T) is associated with an increased risk of renal overload gout in Japanese men only (women not included in this study) [116]	[93,94,100,101]
*SLC17A1*	NPT1	UA secretion in the renal proximal tubule [77,78]	• Major alleles for rs1165196, rs1179086, and rs3757131 associated with higher UA levels in both sexes, where Japanese men also demonstrate high homocysteine and low folic acid levels, while women do not [117]	[82,93,94,96,97,99,101,108]
*SLC22A7*	OAT2	Organic anion/dicarboxylate exchanger located in the renal proximal tubule [118], potential role in UA secretion [119]	• No sex differences reported in humans	[99,101]
*SLC22A11*	OAT4	UA excretion [83]	• Common variant rs17300741 associates with renal underexcretion type gout in Japanese men only (women not included in this study) [83]	[93,94,100]
*SLC22A12*	URAT1	UA reabsorption [81]	• SNP rs893006 major allele associates with higher SUA levels in Japanese men, but not women [120] • SNP rs506338 shows marginally significant association with SUA in Chinese women but not men [114]	[92,93,94,97,100,101,108,112]
*PDZK1*	PDZK1	Scaffold protein, stabilizes UA transporters in the apical compartment [121]	• Variant rs1471633 is associated with increased gout risk in European men but not women [100]	[93,94,96,100,101]
*HNF4A*	HNF4α	Can directly regulate expression of UA transporters *ABCG2* [101] and *SLC2A9* [122]	• Increased activity in the pancreas [123] and liver [107] of women as compared to men	[99,101]
*HNF4G*	HNF4ᵧ	Associations with pro-inflammatory response [124]	• No sex differences reported	[82,94,96,99,100,101]

^1^ Studies that demonstrate gene associations with SUA are referenced.

## Abbreviations

CKD	Chronic Kidney Disease
ER	Estrogen Receptor
ESRD	End Stand Renal Disease
FEUA	Fractional Excretion of Urate
FTM	Female-to-Male
GFR	Glomerular Filtration Rate
GWAS	Genome-Wide Association Studies
HUA	Hyperuricemia
MTF	Male-to-Female
OAT	Organic Anion Transporter
SNP	Single Nucleotide Polymorphism
SUA	Serum Urate
UA	Urate
UUE	Urinary Excretion of Urate
WES	Whole Exome Sequencing
